# Restricted cement augmentation in unstable geriatric midthoracic fractures treated by long-segmental posterior stabilization leads to a comparable construct stability

**DOI:** 10.1038/s41598-021-03336-2

**Published:** 2021-12-10

**Authors:** Ulrich J. Spiegl, Martin Weidling, Viktoria Nitsch, Robin Heilmann, Martin Heilemann, Toni Wendler, Stefan Schleifenbaum, Martin Reinhardt, Christoph-E. Heyde

**Affiliations:** 1 Department of Orthopaedic, Trauma and Plastic Surgery, University Hospital Leipzig, Liebigstr. 20, 04103 Leipzig, Germany; 2 Center for Research On Musculoskeletal Systems (ZESBO), Faculty of Medicine, University of Leipzig, Leipzig, Germany; 3 Department of Diagnostic and Interventional Radiology, University Hospital Leipzig, Leipzig, Germany

**Keywords:** Preclinical research, Trauma

## Abstract

The goal of this study is to compare the construct stability of long segmental dorsal stabilization in unstable midthoracic osteoporotic fractures with complete pedicle screw cement augmentation (ComPSCA) versus restricted pedicle screw cement augmentation (ResPSCA) of the most cranial and caudal pedicle screws under cyclic loading. Twelve fresh frozen human cadaveric specimens (Th4–Th10) from individuals aged 65 years and older were tested in a biomechanical cadaver study. All specimens received a DEXA scan and computer tomography (CT) scan prior to testing. All specimens were matched into pairs. These pairs were randomized into the ComPSCA group and ResPSCA group. An unstable Th7 fracture was simulated. Periodic bending in flexion direction with a torque of 2.5 Nm and 25,000 cycles was applied. Markers were applied to the vertebral bodies to measure segmental movement. After testing, a CT scan of all specimens was performed. The mean age of the specimens was 87.8 years (range 74–101). The mean T-score was − 3.6 (range − 1.2 to − 5.3). Implant failure was visible in three specimens, two of the ComPSCA group and one of the ResPSCA group, affecting only one pedicle screw in each case. Slightly higher segmental movement could be evaluated in these three specimens. No further statistically significant differences were observed between the study groups. The construct stability under cyclic loading in flexion direction of long segmental posterior stabilization of an unstable osteoporotic midthoracic fracture using ResPSCA seems to be comparable to ComPSCA.

## Introduction

Cement augmentation of pedicle screws is one of the most effective strategies to improve pedicle screw stability in osteoporotic vertebral bodies^1–3^. However, the technique is associated with some serious complications. A 30-day mortality rate of almost 2% has been reported^4^. Thus, cement augmentation has to be critically questioned for each individual screw with regard to risks and benefits. This applies particularly to long segmental posterior stabilization, which provides a better load distribution compared to short segmental constructs^2^. Thereby, it can be assumed that the stress level at the screw-bone interface is greatest at the most cranial and most caudal screws. This represents the transition between the rigid instrumented and the freely flexible spine. For this reason, it can be presumed that restricted cementation of long segmental posterior stabilization limited to the most cranial and most caudal vertebrae may achieve comparable stability compared to complete pedicle screw cement augmentation. Restricting cement application can effectively reduce the complication rate. Additionally, it can help to decrease implant cost and surgery time.

Just recently, Spiegl et al.^5^ have found no differences in the maximum force between restricted pedicle screw augmentation and complete pedicle screw augmentation after long segmental stabilization of an unstable midthoracic fracture. The authors concluded that the construct stability of both strategies was comparable under static axial compression. However, this needed to be evaluated by cyclic biomechanical testing.

Based on this, a biomechanical study was performed to compare the construct stability between restricted pedicle screw augmentation and complete pedicle screw augmentation under cyclic loading. We hypothesized that there would be no significant differences between the two groups with respect to segmental movement and signs of screw loosening.

## Materials and methods

### Specimen preparation

Twelve fresh human donor spinal columns (Th4–Th10) were already examined in a previous study performing load of failure testing in flexion direction without visible implant failure or implant loosening in the CT performed after testing^5^. All donors originated from the Institute of Anatomy of the Leipzig University and had given written informed consent to dedicate their bodies to medical education and research purposes. According to the Saxonian Death and Funeral Act of 1994, this study is exempt from the need for approval by a registered ethics committee, however institutional approval for the use of the post-mortem tissues of human body donors was obtained prior to commencing this research. The authors declare that all experiments were performed according to the ethical principles of the Declaration of Helsinki. After computed tomography (CT) and dual-energy X-ray absorption (DEXA) measurements, the intact specimens were grouped into pairs according to gender, mineral bone density, and age. The first group represents the study group with restricted pedicle screw cement augmentation (ResPSCA), while the second group is the control group with complete pedicle screw cement augmentation (ComPSCA) (Table 1).

**Table 1:** Comparison groups with donor characteristics.

Specimen*	Gender	Donor Age (y)	Donor Weight (kg)	Donor Height (m)	Pathology	T-Score
ResPSCA 1	Male	92	84	1.74	Th6 fract	− 4.1
ResPSCA 2	Female	88	51	1.54	Mb Bech	− 4.4
ResPSCA 3	Female	101	39	1.52		− 4.4
ResPSCA 4	Male	90	76	1.65		− 1.9
ResPSCA 5	Male	79	123	1.81		− 1.2
ResPSCA 6	Female	96	74	1.56		− 4.4
Mean value + SD		91 ± 8	75 ± 29	1.6 ± 0.1		− 3.4 ± 1.5
Range		79–101	39–123	1.5–1.8		− 4.4 to − 1.2
ComPSCA 1	Male	74	84	1.74		− 4.0
ComPSCA 2	Female	78	57	1.54		− 4.7
ComPSCA 3	Female	85	54	1.56		− 5.3
ComPSCA 4	Male	93	64	1.69		− 3.1
ComPSCA 5	Male	80	120	1.76	Mb Bech	− 2.0
ComPSCA 6	Female	97	76	1.59		− 3.2
Mean value + SD		85 ± 9	76 ± 24.4	1.6 ± 0.1		− 3.7 ± 1.2
Range		74–97	54–120	1.5–1.8		− 5.3 to − 2.0
*p* values^#^		0.20	0.93	0.88		0.75

ResPSCA: cement augmentation of pedicle screws at cranial (Th5) and caudal (Th9) level only; ComPSCA: cement augmentation of all pedicle screws; Mb Bech: Morbus Bechterew; Th6 Fract: Consolidated fracture of Th 6; SD: Standard Deviation.

*For a pairwise comparison between the groups, specimen pairs were assigned the same specimen number.

^#^Statistical evaluation of mean value differences between the groups, *p* values < 0.05 stating significant difference.

Soft tissues were dissected while preserving intervertebral discs, facet joints and ligament structures. Based on the CT scans, appropriate pedicle screw diameters and optimum screw lengths were determined. All pedicles of the levels Th5, Th6, Th8 and Th9 were instrumented transpedicularly using cannulated pedicle screws (M.U.S.T., Medacta Corporate, Switzerland) according to the manufacturer's specifications. This included the identification of the correct entry point, which was opened with a sharp awl, predrilling with a 2.5 mm drill bit, subsequent tapping, checking for intactness using a pedicle button, and insertion of the pedicle screws parallel to the cover plate with approximately 10° convergence. Cement augmentation of the pedicle screws was performed according to the previously defined groups. In the study group, cement augmentation was performed at the pedicle screws of the cranial (Th5) and caudal (Th9) level only (ResPSCA). All pedicle screws were cement augmented in the control group (ComPSCA). The bone cement (In. Medtronic, USA) was mixed and 1.0 ml of bone cement was applied slowly over each cemented cannulated screw using a cement application syringes. After curing of the bone cement, the cementing instruments were removed. Afterwards, two titanium rods were contoured to the coronary and sagittal alignment of the respective body donor and the rods were fixed to the pedicle screws according to the manufacturer's instructions. An unstable fracture was modeled by resection of a standardized ventral wedge of Th7 including the posterior cortex. For reproducibility, Kirschner wires were placed over a template to guide the osteotomy^5^.

The specimens were compressed axially and eccentrically by 20 mm. In a subsequent CT evaluation, no screw loosening and no damage to the spinal column structure was found^5^. All specimens were wrapped in plastic foil, cooled and shock frozen at − 80 °C to minimize ice crystal growth^6^.

For the current study, the specimens were then gently thawed. The temperature was gradually increased, for at least 2 days, to − 20 °C, then for one day to − 2 °C, then transitioned to room temperature within 16 h prior to testing. This is intended to reduce the temperature gradient within the specimen during thawing and to protect the tissues.

### Experimental procedure

The non-instrumented vertebrae Th4 and Th10 were embedded with a polyurethane casting resin (RenCast; Huntsman Advanced Materials, Basel, Switzerland). Additional screws were inserted into the vertebral bodies to improve the bond between the bone and embedding. The vertebral endplate of Th7 was positioned horizontally to ensure an upright alignment of the spine.

**Figure 1:**
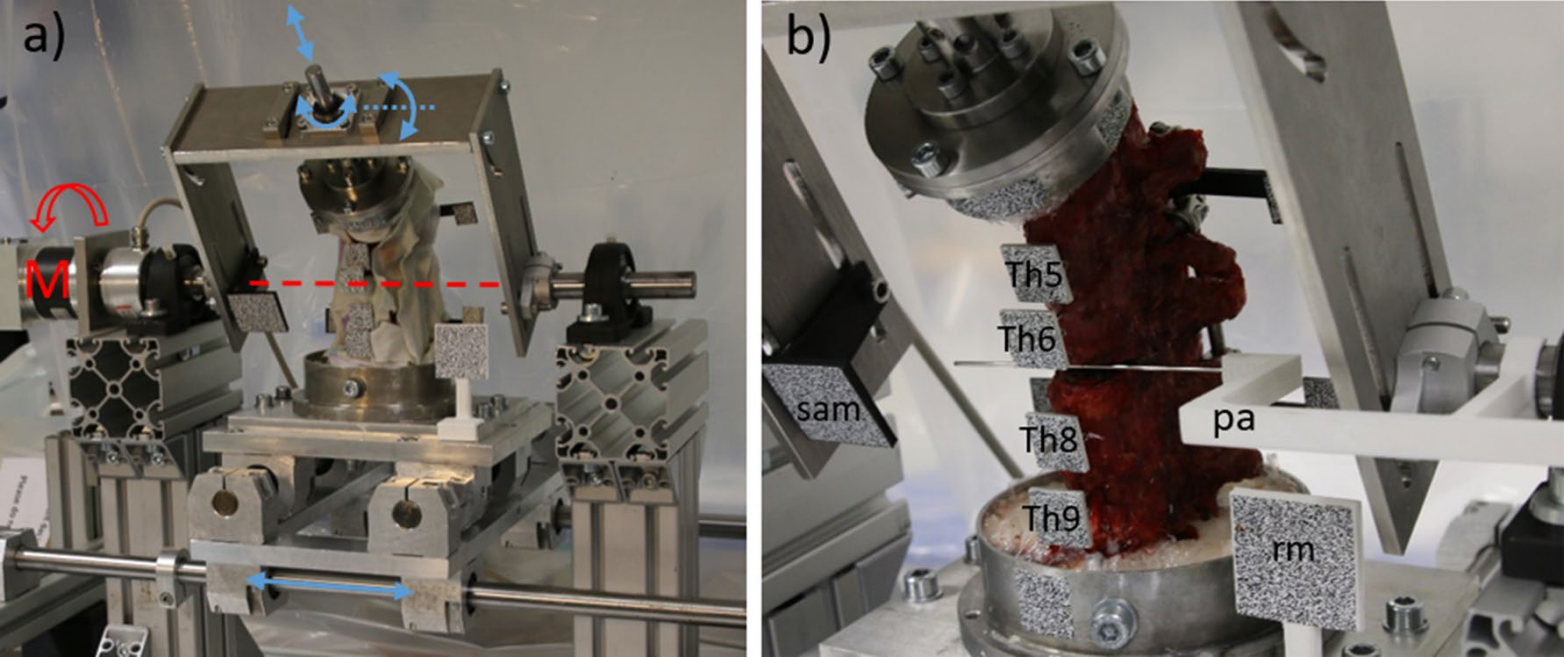
Experimental set-up: (**a**) test stand: a torque around the axis of the swivel arm (red line) is introduced into the specimen via the motor (M), the blue arrows represent the degrees of freedom of the clamped specimen; (**b**) Markers with speckle patterns are applied to the instrumented vertebral bodies (Th5, Th6, Th8 and Th9), the swivel arm (sam) and a reference marker (rm). In addition, a positioning aid (pa) for aligning the axis of rotation to the fracture gap is shown.

The specimens were clamped in a test stand developed in-house (Fig. 1a). The major component is a swivel arm that is driven by a motor, generating a defined torque. The specimens were fixed with the lower embedding on a slide, while the upper embedding was connected to the swivel arm. The rotation axis of the swivel arm was set to the center of the fracture gap in Th7 (Fig. 1b). In order to generate torque as straight as possible into the spinal column, the specimen was not fully constrained (Fig. 1a). The slide allowed lateral movements while forward and backward movements were suppressed. The upper embedding was connected via a bearing rod to a linear bearing in the swivel arm. This enabled rotation and axial compensatory movements of the spinal column. The linear bearing was, in turn, pivotally mounted in the swivel arm. Thereby, mainly torque in the flexion/extension direction was introduced, whereas pure transverse forces were minimized.

Markers with speckle patterns were pinned to the instrumented vertebral bodies (Fig. 1). The pins were pinned into the vertebral body. Care was taken to ensure that they were far away from the screw tips and the surrounding cement, which was introduced after carefully evaluating the CT scan after compressive testing. Markers were also attached to the swivel arm, as well at an independent reference point.

The specimens were periodically bent in the direction of flexion. A torque of 2.5 Nm was applied, as recommended in osteoporotic thoracic spines^7^. A total of 25,000 load cycles were applied, which corresponds to the expected motion within the first 3–4 weeks after surgery in a geriatric patient population^8^. Tests were carried out at a frequency of 1.2 Hz. During a load cycle, the load was applied in the first half and released in the second half. The specimens were kept moist throughout the testing period, being wrapped in moist gauzes that were regularly moistened^7^. The rotation of the swivel arm was measured with an angle sensor (Incremental encoder 5821, Fritz Kübler GmbH, Germany). Furthermore, the positions of the markers were recorded with a digital image correlation system with a three-camera setup (Q400, LIMESS Messtechnik und Software GmbH, Krefeld, Germany)^9^. These measurements were taken at the beginning (10 cycles), every 500 cycles and at the end (24,990 cycles) for continuous monitoring. Two cycles were recorded with a frame rate of 15 Hz for each individual measurement time-point.

### Evaluation

After cyclic loading, CT was performed in order to detect any signs of implant failure, screw loosening or subsequent vertebral fractures. These were evaluated independently by two of the authors, one spine surgeon (U.J.S.) and one radiologist (M.R.).

As the markers are anchored into vertebral bodies, it is assumed that they represent the movement of the vertebral bodies during loading^9^. The marker positions measured with the digital image correlation system were correlated and exported into a coordinate system corresponding to a person standing upright. An evaluation routine was developed to calculate the relative movement between two markers. Since torque was introduced, the evaluation was limited to the relative rotation of the vertebral bodies. In order to calculate these rotational components about a respective axis of the coordinate system, one vector defined by two speckle pattern points on each marker, respectively, was regarded. When selecting these points, it was ensured that the resulting vector was preferably perpendicular to the respective rotation axis prior to loading. For all three axes of the coordinate system, the projection of the respective vector into the plane perpendicular to the respective axis was regarded. The angles between two vector projections of different markers were calculated for each time step. Thus, relative rotation depending on the regarded axis could be calculated for any pair of markers. The calculation was done using MATLAB (MathWorks and Simulink, USA).

The relative rotations between the swivel arm and the reference marker were compared with the data from the angle sensor to check the continuity of the measurements (Supplement). The relative rotations between the adjacent vertebrae Th5/Th6 and Th8/Th9 and between the vertebra pairs Th5/Th9 and Th6/Th8 were evaluated. For each time interval of a series of measurements, the peak-to-peak amplitude and the zero offset to the rest position were determined. In the course of the measurement, the part of the movement characterized by the peak-to-peak amplitude was regarded as reversible. A non-reversible part was indicated by the difference between the zero offset and the rest position of the first time interval. This was subsequently defined as permanent deflection (Supplement). The determined permanent deflections and peak-to-peak amplitudes were considered separately and examined in the course of the 25,000 cycles.

The statistical analysis was performed with SPSS 24.0 (IBM, USA). The Shapiro–Wilk test was used to verify normal distribution. Mean differences were checked with the Student t-test for normally distributed data pairs, otherwise the Mann–Whitney test was used. A value of *p* < 0.05 was considered significant.

## Results

**Figure 2:**
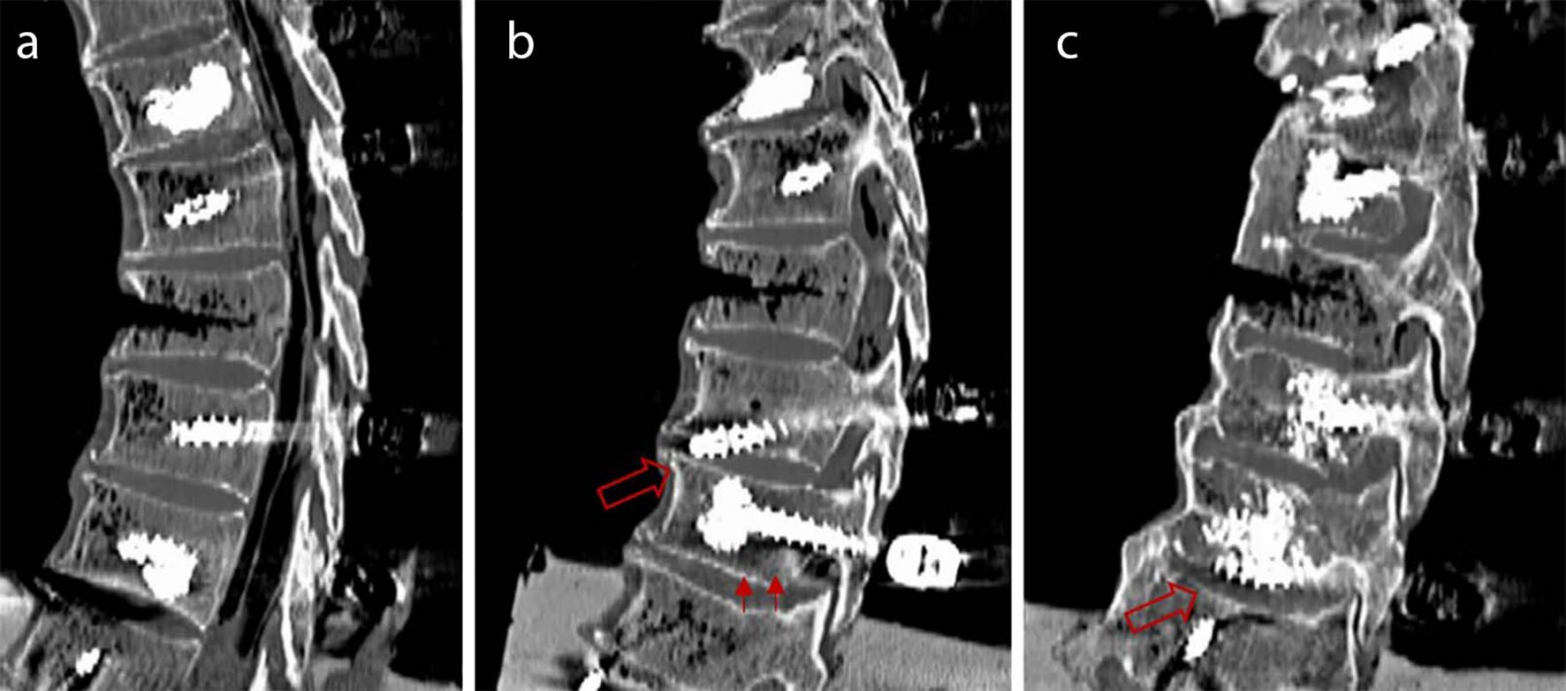
CT scans after cyclic loading are illustrating one of five cases with ResPSCA, without any signs of screw loosening or implant failure (**a**), one specimen with cut-out of the right pedicle screw in Th8 (big arrow) and signs of loosening of the right cement-augmented pedicle screw in Th9 (**b**, small arrows). In (**c**), one of two specimens is shown with a cut-out of the right cement-augmented pedicle screw of Th9 after ComPSCA (arrow).

Evaluation of the CT images showed loosening of pedicle screws in three specimens, including screw loosening in one specimen of the study group (Fig. 2a–c). Thereby, a cut-out of the right pedicle screw in Th8 and some signs of loosening of the right augmented pedicle screw in Th9 of specimen ResPSCA 1 were visible (Fig. 2b). In the control group, screw loosening was observed in two specimens. Screw cuts out of the right pedicle screws in Th9 could be seen in ComPSCA 2 and ComPSCA 5 (Fig. 2c).

In all specimens, mainly relative rotations around the transverse axis were observed, on which the assessment focuses. The marker in Th5 of the specimen ComPSCA 2 protruded slightly into the disc. Based on a potential effect on the measurements, this marker was not taken into account in the assessment. During loading, no pronounced periodic movement between the adjacent vertebral bodies was observed. In the course of the measurements over all specimens, no abrupt changes were detected, which would have indicated premature failure. For this reason, only cycles 10, 5,000, 10,000, 15,000, 20,000 and 24,990 were evaluated.

**Figure 3:**
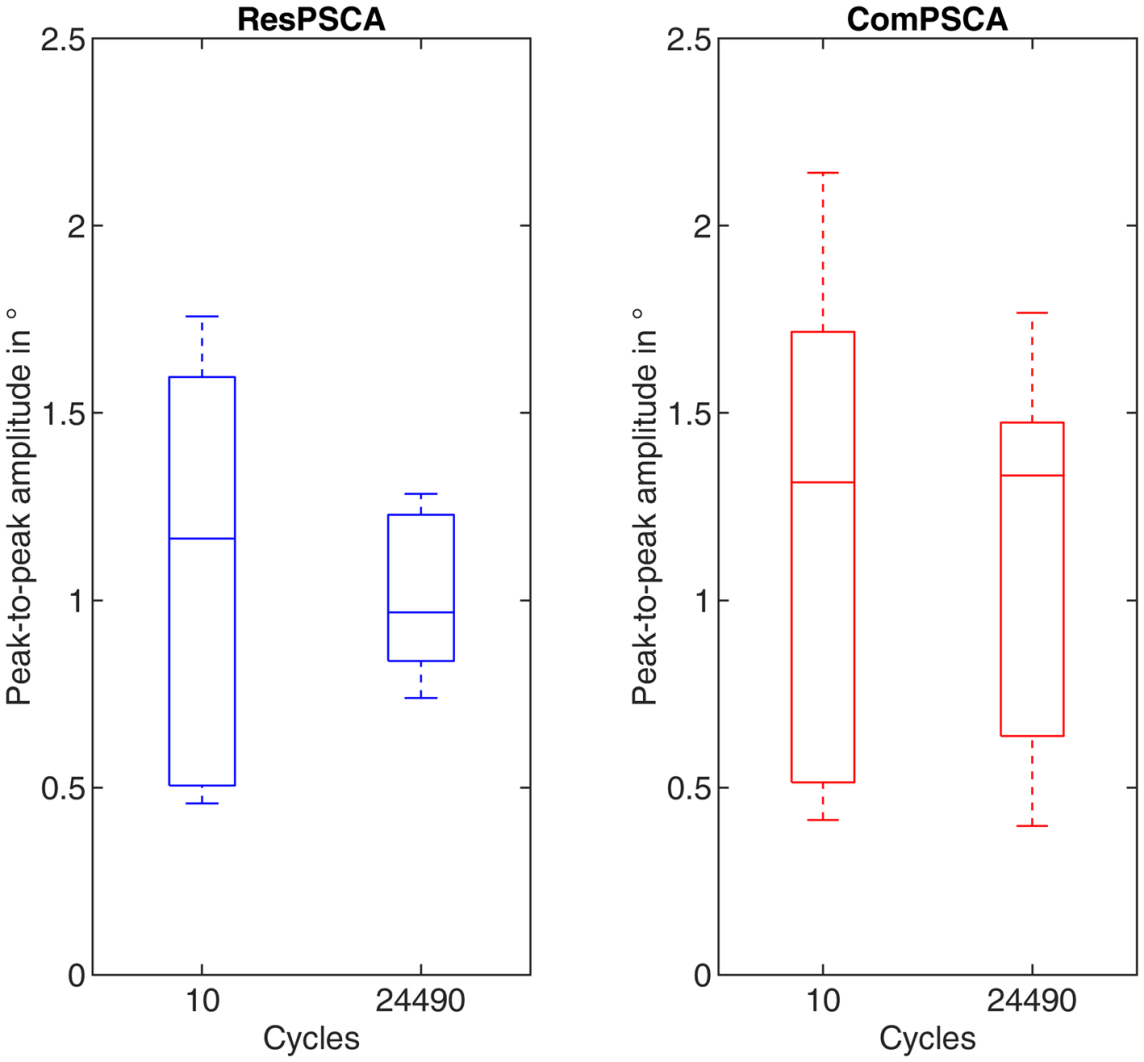
Comparison of the peak-to-peak amplitudes between Th6 and Th8 at the beginning (cycle 10) and end (cycle 24,490) of the testing for restricted cement augmentation (ResPSCA) and complete cement augmentation (ComPSCA). Statistically, there are no differences in the mean values of the peak-to-peak amplitudes between the beginning and the end of testing in between a group (*p* = 0.67 for ResPSCA, *p* = 0.83 for ComPSCA) and in the direct comparison between both groups (*p* = 0.73 for cycle 10 between ResPSCA and ComPSCA, *p* = 0.53 for cycle 24,490 between ResPSCA and ComPSCA).

In Fig. 3, box plots of the peak-to-peak amplitudes between Th6 and Th8, at the beginning and end of the measurements, of both study groups are shown. Statistically, there were no differences in the mean values of the peak-to-peak amplitudes between the beginning and the end of testing in between the groups (*p* = 0.67 for ResPSCA, *p* = 0.83 for ComPSCA), as well as between both groups (*p* = 0.73 for cycle 10 between ResPSCA and ComPSCA, *p* = 0.53 for cycle 24,990 between ResPSCA and ComPSCA).

**Figure 4:**
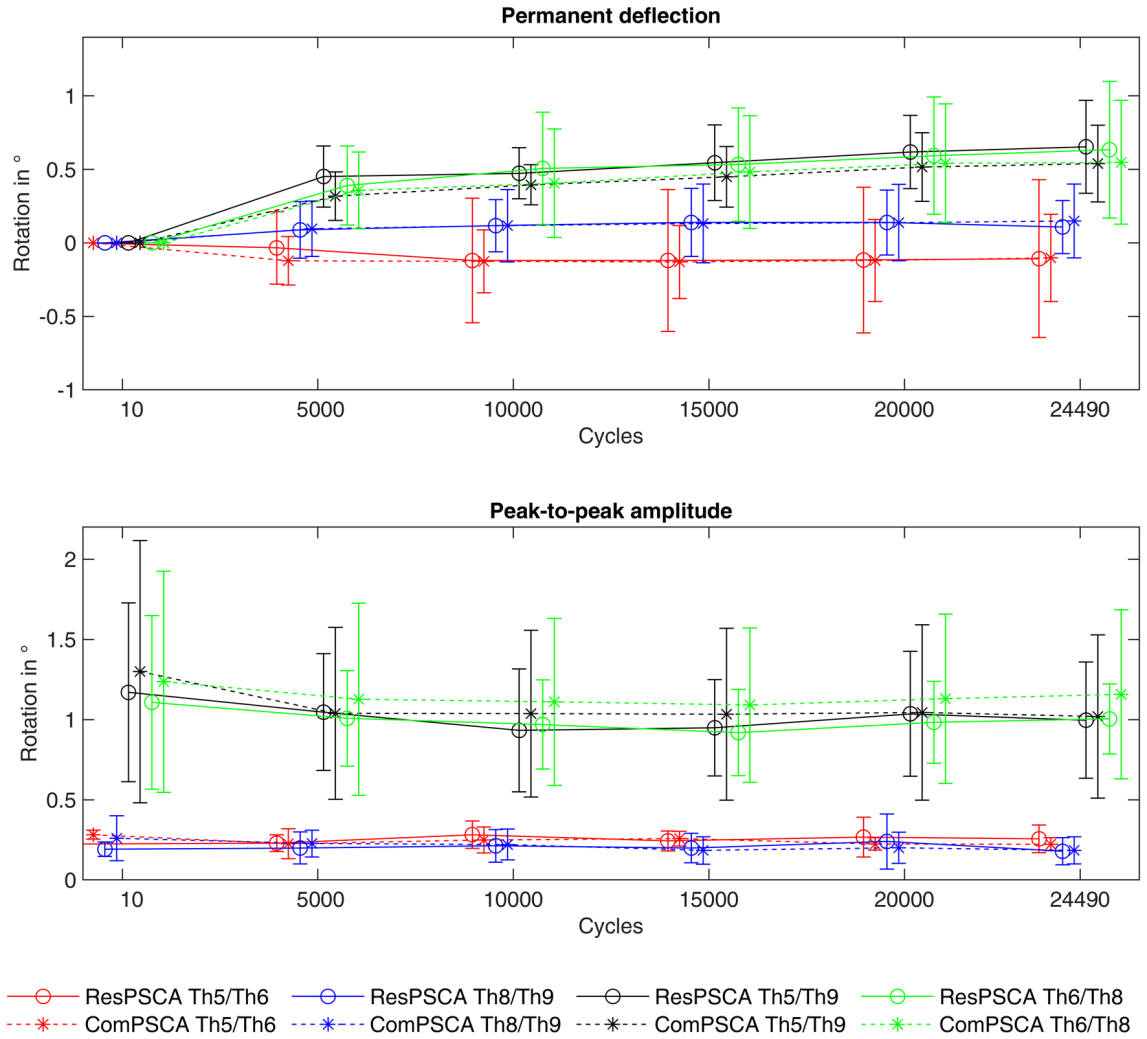
Comparison of the mean values of the test groups with complete (ComPSCA) and restricted cement augmentation (ResPSCA) with regard to permanent deflection (above) and peak-to-peak amplitude (below). No statistically significant differences were found when comparing any pair of data for the ResPSCA and ComPSCA groups. To make the error bars more visible, the dots have been slightly shifted. However, the measured values refer to the cycle indicated on the abscissa.

Figure 4 compares the mean values of the calculated permanent deflections and peak-to-peak amplitudes for both test groups with complete (ComPSCA) and restricted cement augmentation (ResPSCA). For each of the vertebral body pairs the course of the measured values between the two comparison groups appeared to be qualitatively and quantitatively similar. This finding is supported by the fact that, for each data pair between the ResPSCA and ComPSCA groups, the mean values were examined and no statistically significant differences were found (Table 2).

**Table 2:** Comparison of the mean values of the test groups with complete (ComPSCA) and restricted cement augmentation (ResPSCA) with regard to permanent deflection and peak-to-peak amplitude.

Cycles	5000	10,000	15,000	20,000	24,490	
**Permanent deflection (in °)**						
**Th5/Th6**						
ResPSCA*	− 0.03 ± 0.25	− 0.12 ± 0.42	− 0.12 ± 0.48	− 0.12 ± 0.50	− 0.11 ± 0.54	
ComPSCA*	− 0.12 ± 0.17	− 0.13 ± 0.21	− 0.13 ± 0.28	− 0.12 ± 0.28	− 0.10 ± 0.30	
*p* value^#^	0.27	0.36	0.36	0.58	0.72	
**Th8/Th9**						
ResPSCA*	0.09 ± 0.19	0.12 ± 0.18	0.14 ± 0.23	0.14 ± 0.22	0.11 ± 0.18	
ComPSCA*	0.10 ± 0.19	0.12 ± 0.25	0.13 ± 0.27	0.14 ± 0.26	0.15 ± 0.25	
*p* value^#^	0.75	0.99	0.63	0.99	0.52	
**Th5/Th9**						
ResPSCA*	0.45 ± 0.21	0.47 ± 0.17	0.55 ± 0.26	0.62 ± 0.25	0.65 ± 0.32	
ComPSCA*	0.32 ± 0.16	0.40 ± 0.14	0.45 ± 0.21	0.52 ± 0.23	0.54 ± 0.26	
*p* value^#^	0.27	0.43	0.52	0.50	0.53	
**Th6/Th8**						
ResPSCA*	0.39 ± 0.27	0.51 ± 0.38	0.53 ± 0.39	0.59 ± 0.40	0.63 ± 0.46	
ComPSCA*	0.36 ± 0.26	0.41 ± 0.37	0.48 ± 0.38	0.54 ± 0.40	0.55 ± 0.42	
*p* value^#^	0.83	0.65	0.82	0.83	0.74	

Cycles	10	5000	10,000	15,000	20,000	24,490
**Peak-to-peak amplitude (in °)**						
**Th5/Th6**						
ResPSCA*	0.22 ± 0.10	0.23 ± 0.05	0.28 ± 0.09	0.24 ± 0.06	0.27 ± 0.12	0.26 ± 0.09
ComPSCA*	0.28 ± 0.03	0.23 ± 0.09	0.25 ± 0.08	0.26 ± 0.05	0.22 ± 0.04	0.22 ± 0.04
*p* value^#^	0.27	0.94	0.53	0.69	0.48	0.86
**Th8/Th9**						
ResPSCA*	0.19 ± 0.05	0.20 ± 0.10	0.21 ± 0.10	0.20 ± 0.09	0.24 ± 0.17	0.18 ± 0.08
ComPSCA*	0.26 ± 0.14	0.23 ± 0.08	0.22 ± 0.10	0.18 ± 0.9	0.20 ± 0.10	0.19 ± 0.08
*p* value^#^	0.28	0.61	0.89	0.77	0.64	0.89
**Th5/Th9**						
ResPSCA*	1.17 ± 0.56	1.05 ± 0.36	0.93 ± 0.38	0.95 ± 0.30	1.04 ± 0.39	1.00 ± 0.36
ComPSCA*	1.3 ± 0.82	1.04 ± 0.54	1.04 ± 0.52	1.03 ± 0.54	1.04 ± 0.55	1.02 ± 0.51
*p* value^#^	0.76	0.98	0.71	0.75	0.98	0.93
**Th6/Th8**						
ResPSCA*	1.11 ± 0.54	1.01 ± 0.30	0.97 ± 0.28	0.92 ± 0.27	0.98 ± 0.25	1.00 ± 0.22
ComPSCA*	1.23 ± 0.69	1.13 ± 0.60	1.11 ± 0.52	1.09 ± 0.48	1.13 ± 0.53	1.16 ± 0.53
*p* value^#^	0.73	0.67	0.57	0.46	0.55	0.53

ResPSCA—pedicle screws at most cranial (Th5) and most caudal (Th9) are cement augmented, ComPSCA—all pedicle screws are cement augmented.

*Measured values given as mean value ± standard deviation (in degree).

^#^Statistical analysis performed, stating significant difference between mean values for compared groups at *p* < 0.05.

**Figure 5:**
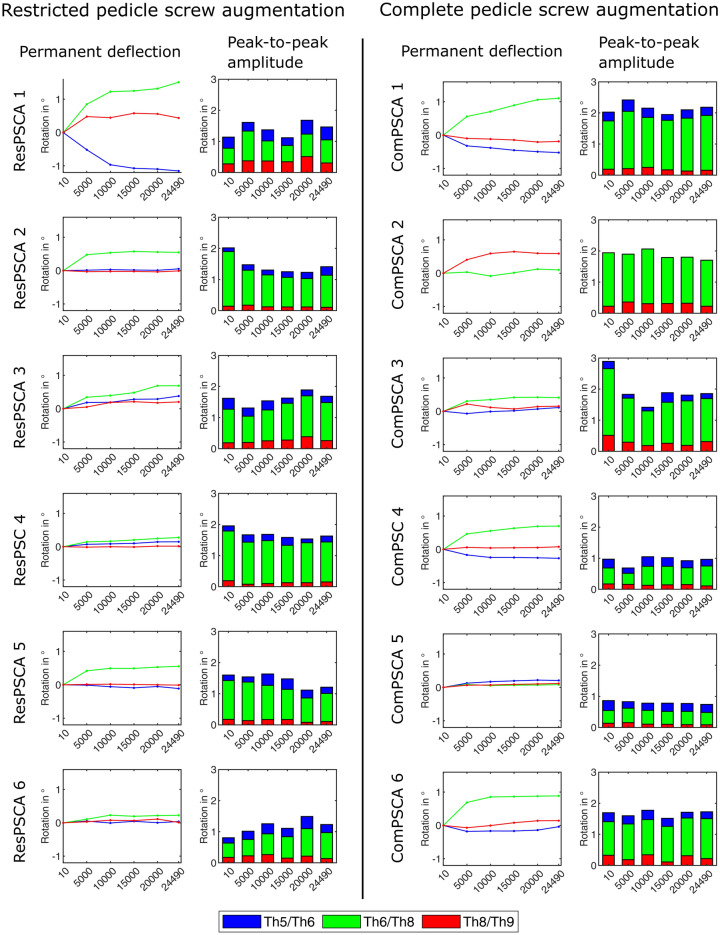
Comparison of the permanent deflections and peak-to-peak amplitudes of the observed vertebral body pairs of both experimental groups against performed cycles. With ComPSCA 2, the values for Th5/Th6 had to be excluded.

Figure 5 compares the two test groups. In each case, the permanent deflections and peak-to-peak amplitudes of the comparison pairs Th5/Th6, Th6/Th8 and Th8/Th9 are considered. In most cases, the permanent deflections of Th5/Th6 and Th8/Th9 were small and comparatively smaller than those between Th6/8, with the exception of ResPSCA 1, ComPSCA 2 and ComPSCA 5, respectively. In all of those three specimens, implant failure was visible. The peak-to-peak amplitudes of Th5/Th6 and Th8/Th9 were significantly smaller than those of Th6/Th8, but the differences were less obvious in the specimens ResPSCA 1, ResPSCA 6, ComPSCA 4, and ComPSCA 5.

## Discussion

The most important finding of this article is the comparable construct stability between ComPSCA and ResPSCA with two cases of cut-outs in the ComPSCA group and only one in the ResPSCA group under cyclic testing, despite the fact that biomechanical testing under axial loading was done previously in all specimens. The dynamic testing results confirm these three cases of implant failure. Hereby, the orientation of two vertebrae has changed permanently during the course of cyclic loading, which can be interpreted as a sign of screw loosening. However, based on the fact that only a one-sided screw cut-out was seen in all three cases, with signs of implant failure and macroscopically uneventful contralateral screw positioning, no higher grades of instability can be expected. This is in accordance with our results, with consistent but only subtle differences in segmental movement between the three specimens with implant failure in comparison to the others.

Otherwise the peak-to-peak amplitudes of movement were in accordance with the expected results. Minimal to low peak-to-peak amplitudes were recorded in the stabilized healthy segments Th5/Th6 and Th8/Th9. In contrast, moderate to high peak-to-peak amplitudes were seen between Th6/Th8, which represents the stabilized unstable fracture region. Generally, the ranges of peak-to-peak amplitudes between the specimens were large without any significant differences between the study groups. This seems to be not very surprising considering the rather small study group and the big range of ages in patients and the morphological differences between the spines. However, both study groups were matched regarding patient age, bone density and gender in order to minimize the differences between the groups.

Interestingly, two of the specimens with implant failure were highly osteoporotic, with T-scores of less than -4 (two cases). The third specimen had spondylitis ancylosans. Several authors recommend long segmental stabilization with pedicle screw implantation of three levels above and below the fracture in patients with spondylitis ancylosans^10,11^. This can partially explain the implant failure. However, all implant failure happened to be below the fracture. This is somewhat surprising, as in daily practice screw cut-outs seem to occur more frequently in the instrumented vertebral bodies above the fracture in correspondence to the data reported by Banno et al.^12^. In contrast, other studies reported observed higher rates of screw loosening at the lowest level of instrumentation^13^. Generally, the huge majority of implant failure occurs at the lowest or highest level of instrumentation^12,13^. Additionally, all cut-outs were one-sided. This can be explained by the fact that the cascade of implant failure has just begun. This might end in screw cut-outs of both pedicle screws, leading to higher instabilities in the further course.

Generally, specimens tended to adopt a proceeding kyphotic malposition during the course of testing due to cyclic loading of 25,000 cycles predominantly in the flexion direction. The permanent deflection was expected based on cyclic loading without protective interactions of the muscle and rib cage due to permanent strain on the connective tissues. Generally, 25,000 cycles represent the average load during all day activities over a period of 3–4 weeks for elderly people^8^. This number of cycles was chosen to simulate this very important period of bony healing. In correspondence to that, increased in vivo stiffness has been observed to begin 3 weeks after osteotomy in an ostoporotic sheep model^14^. In addition, fatigue tests should be conducted in follow-up studies to evaluate the long-term behavior of the stabilization. Thus, the load acting on the material can be supposed to be higher as compared to clinical practice. The selected bending moment of 2.5 Nm is based on a literature recommendation for range of motion tests on osteoporotic thoracic spines as maximal loads in order not to destroy tissues^7^. In vivo tests of the more heavily loaded lumbar spine measured 3.5 (± 1.5) Nm when bending the upper body and 4.2 (± 1.7) Nm when lifting a weight from the floor^15^. On the one hand, significantly lower loads are assumed to be in the area of the middle thoracic spine. On the other hand, upper body flexion and weight lifting are extreme loads that should be avoided postoperatively. By performing cyclic testing over an estimated period of 3 to 4 weeks and applying high cyclic loads, a model was chosen that simulates an extreme situation without any stabilizing effect that would be expected in living patients as a part of the fracture healing process.

When evaluating the relative movement between the individual vertebral bodies, indications of screw loosening were found, but there were no clear patterns. An indirect measuring method was chosen, which allows for continuous observation. In order to measure screw movement in the vertebral body directly, markers would have to be attached to the screw tip or shaft. This would require the removal of bone material, which would have a lasting effect on screw retention. This was not the intention of the study, but should be investigated in subsequent studies.

However, the study has several limitations. First of all, all specimens were previously tested in a load of failure manner by axial compression. Thereby, implant failure particularly screw cut-out or screw loosening could be excluded by CT examination after testing^5^. A large part of the deformation was elastically stored in the rod system through the fracture gap. Despite the fact that it is not possible to definitely exclude minor lesions, only a minority of specimens showed signs of implant failure. Generally, all specimens had a similar load history and were appropriate for a comparative study. Secondly, another freezing and thawing cycle can influence the mechanical properties of soft tissue negatively^16^. However, the influence on the mechanical properties of bone tissue seems to be not relevant^17,18^. Furthermore, only minor effects on the range of motion of functional spine units have been observed^19^. In a further study, several freezing and thawing cycles were examined. No significant alterations in the range of motion could be seen after the initial freezing during further freeze–thaw cycles^20^. In addition, the samples were frozen in a tissue-friendly manner^6^. As the samples have the same storage history, comparative studies are permissible. In addition, the study focuses on the screw anchorage in the bone. The relevant vertebral bodies are rigidly instrumented. The freely movable segments, on which alterations of the intervertebral discs and ligaments would have a greater impact, were not the focus of this study. For the reasons mentioned above, a comparative study with the specimens is permissible, even though they have already undergone initial testing. Since all specimens were always treated in the same way, comparability is ensured. In addition, the usual recommendations were followed for storage, test duration, moisture retention, load rates, etc.^7,21,22^. Additionally, the cyclic loading was performed in flexion only. In contrast, human spines are subjected to multiple different loadings in different directions, all of which contribute to the development of implant failure. Thereby, the midthoracic spine is particularly susceptible under flexion with lower flexion strength than compressive strength^23^. Furthermore, it was not possible to generate pure torque only. However, the test set-up applies a uniform torque in the direction of flexion in a reproducible manner, which ensures comparability. Additionally, our sample size was small (six spines in each group) and underpowered. A post-hoc analysis has shown that at least 80 speciment per group are necessary to gain a power of 80%. However, compared with related publications, our study had a similar number of specimens per group^24–27^ and complies with the recommendations for in vitro testing with human donor material^20^. Thereby, the analysis of group differences can be misleading based on the low power. Nevertheless, there were two implant failures visible in the ComPSCA group and only one in the ResPSCA group. Additionally, matching of the groups was performed in accordance to the T-score, age, and gender of the specimen. Next, the anatomic model represents a simplified model not considering the rib cage (leading to a decrease in stiffness), the muscle, and the physiological body weight acting on the midthoracic cage^28,29^. Last but not least, we did not include a non-cemented group in order to prove that cement-augmented pedicle screw augmentation is superior in our testing scenario. This was done based on the moderate to good biomechanical evidence of the superiority cement-augmented screw hold in osteoporotic bone^30,31^. Based on this evidence and the clinical experience of the last decade the authors hardly ever perform posterior stabilization without cement-augmented pedicle screws in osteoporotic vertebral body fractures. Generally, only clinical studies are conclusive for the evaluation of screw loosening in everyday life. Therefore, clinical studies are warranted to compare implant failure and reduction loss between restricted and complete pedicle screw augmentation in long segmental posterior stabilization.

## Conclusion

No statistically significant differences in both implant failure rate and peak-to-peak amplitudes of movement between the instrumented vertebral bodies could be seen between the ResPSCA and ComPSCA groups under cyclic loading. Thus, the construct stability of long segmental posterior stabilization of an unstable osteoporotic midthoracic fracture using ResPSCA seems to be comparable to ComPSCA.

## Supplementary Information

Supplementary Legends.

Supplementary Figures.
