# Supplementary material for: Restricted cement augmentation in unstable geriatric midthoracic fractures treated by long-segmental posterior stabilization leads to a comparable construct stability
Source: Sci Rep. 2021 Dec 10;11:23816. doi: 10.1038/s41598-021-03336-2 (PMC8664925; doi:10.1038/s41598-021-03336-2)
Supplement: Supplementary file 1 — Supplementary Legends. [file 41598_2021_3336_MOESM1_ESM.docx]

**Supplement Figure legends**: Evaluation of the calculated relative rotations: a) Comparison of the measuring points of the angle sensor with the relative rotation between the swivel arm and reference marker determined from the optical measurement; b) Determinations of the peak-to-peak amplitudes and permanent deflections for each time interval of a measurement.
