# Supplementary figures and images for: Restricted cement augmentation in unstable geriatric midthoracic fractures treated by long-segmental posterior stabilization leads to a comparable construct stability
Source: Sci Rep. 2021 Dec 10;11:23816. doi: 10.1038/s41598-021-03336-2 (PMC8664925; doi:10.1038/s41598-021-03336-2)

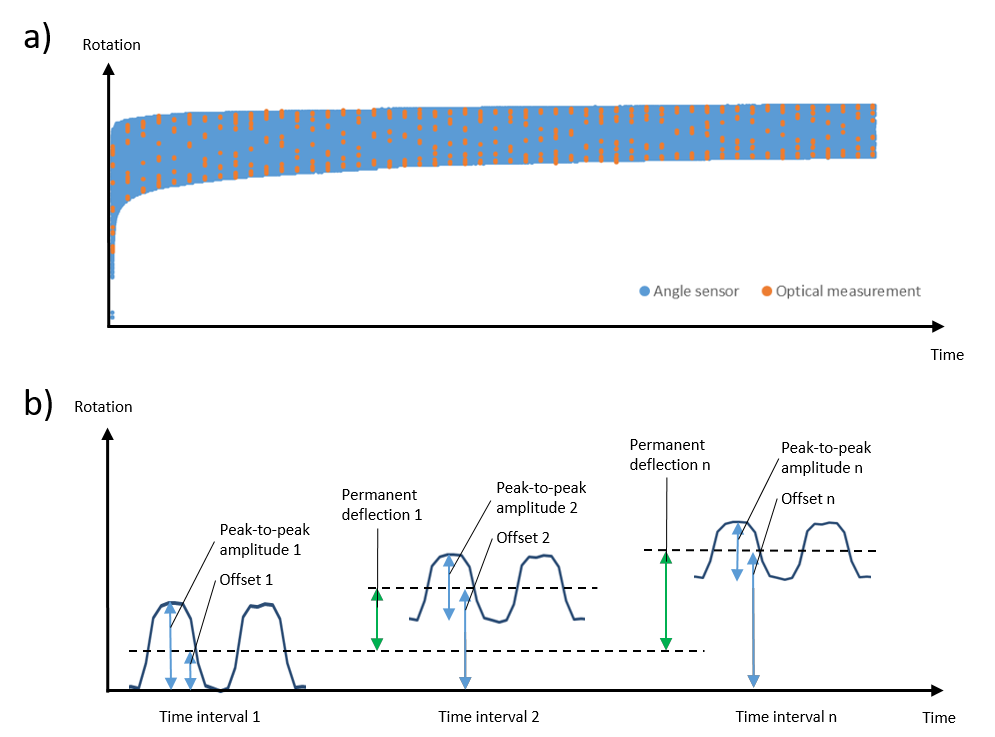

Supplement: Supplementary file 2 — Supplementary Figures. [file 41598_2021_3336_MOESM2_ESM.tif]
